# If we build it, will they come? Results of a quasi-experimental study assessing the impact of maternity waiting homes on facility-based childbirth and maternity care in Zambia

**DOI:** 10.1136/bmjgh-2021-006385

**Published:** 2021-12-06

**Authors:** Nancy A Scott, Jeanette L Kaiser, Thandiwe Ngoma, Kathleen L McGlasson, Elizabeth G Henry, Michelle L Munro-Kramer, Godfrey Biemba, Misheck Bwalya, Viviane R Sakanga, Gertrude Musonda, Davidson H Hamer, Carol J Boyd, Rachael Bonawitz, Taryn Vian, Margaret E Kruk, Rachel M Fong, Parker S Chastain, Kaluba Mataka, Eden Ahmed Mdluli, Philip Veliz, Jody R Lori, Peter C Rockers

**Affiliations:** 1 Department of Global Health, Boston University School of Public Health, Boston, Massachusetts, USA; 2 Department of Research, Right to Care Zambia, Lusaka, Zambia; 3 Biostatistics and Epidemiology Data Analytics Center, Boston University School of Public Health, Boston, Massachusetts, USA; 4 Department of Health Behavior & Biological Sciences, University of Michigan School of Nursing, Ann Arbor, Michigan, USA; 5 National Health Research Authority Zambia, Lusaka, Zambia; 6 Africare Zambia, Lusaka, Zambia; 7 Section of Infectious Diseases, Boston University School of Medicine, Boston, Massachusetts, USA; 8 Center for the Study of Drugs, Alcohol, Smoking and Health, University of Michigan School of Nursing, Ann Arbor, Michigan, USA; 9 University of San Francisco - School of Nursing and Health Professions, San Francisco, California, USA; 10 Department of Global Health and Population, Harvard T. H. Chan School of Public Health, Boston, Massachusetts, USA; 11 Zenysis Technologies, Lusaka, Zambia; 12 Project HOPE, Bethesda, Maryland, USA; 13 Applied Biostatistics Laboratory, University of Michigan School of Nursing, Ann Arbor, Michigan, USA; 14 Center for Global Affairs & PAHO/WHO Collaborating Center, University of Michigan School of Nursing, Ann Arbor, Michigan, USA

**Keywords:** maternal health, intervention study, health services research

## Abstract

**Introduction:**

Maternity waiting homes (MWHs) aim to increase access to maternity and emergency obstetric care by allowing women to stay near a health centre before delivery. An improved MWH model was developed with community input and included infrastructure, policies and linkages to health centres. We hypothesised this MWH model would increase health facility delivery among remote-living women in Zambia.

**Methods:**

We conducted a quasi-experimental study at 40 rural health centres (RHC) that offer basic emergency obstetric care and had no recent stockouts of oxytocin or magnesium sulfate, located within 2 hours of a referral hospital. Intervention clusters (n=20) received an improved MWH model. Control clusters (n=20) implemented standard of care. Clusters were assigned to study arm using a matched-pair randomisation procedure (n=20) or non-randomly with matching criteria (n=20). We interviewed repeated cross-sectional random samples of women in villages 10+ kilometres from their RHC. The primary outcome was facility delivery; secondary outcomes included postnatal care utilisation, counselling, services received and expenditures. Intention-to-treat analysis was conducted. Generalised estimating equations were used to estimate ORs.

**Results:**

We interviewed 2381 women at baseline (March 2016) and 2330 at endline (October 2018). The improved MWH model was associated with increased odds of facility delivery (OR 1.60 (95% CI: 1.13 to 2.27); p<0.001) and MWH utilisation (OR 2.44 (1.62 to 3.67); p<0.001). The intervention was also associated with increased odds of postnatal attendance (OR 1.55 (1.10 to 2.19); p<0.001); counselling for family planning (OR 1.48 (1.15 to 1.91); p=0.002), breast feeding (OR 1.51 (1.20 to 1.90); p<0.001), and kangaroo care (OR 1.44 (1.15, 1.79); p=0.001); and caesarean section (OR 1.71 (1.16 to 2.54); p=0.007). No differences were observed in household expenditures for delivery.

**Conclusion:**

MWHs near well-equipped RHCs increased access to facility delivery, encouraged use of facilities with emergency care capacity, and improved exposure to counselling. MWHs can be useful in the effort to increase delivery at advanced facilities in areas where substantial numbers of women live remotely.

**Trial registration number:**

NCT02620436.

**Key questions**

**What is already known?**

- The WHO recommends that maternity waiting homes (MWHs) may be a useful tool to facilitate access to skilled maternity care, though recognises the evidence base is weak.
- There is limited rigorous quantitative evidence on the effectiveness of MWHs on improving access to quality maternity care, particularly in areas where a large proportion of the population lives remotely.
- A synthesis of qualitative evidence found that low uptake of MWHs in low-income and middle-income countries is driven by a lack of community acceptability, distance, culturally inappropriate care, poor physical infrastructure and a lack of space for companions.

**What are the new findings?**

- The community-informed MWH model tested here significantly increased facility delivery among women living greater than 10 km from their designated rural health centre, directly addressing this distance barrier.
- Secondarily, we observed improvements in MWH utilisation, exposure to maternal and well-baby counselling, and postnatal care attendance, suggesting the benefits of MWHs extend along the care continuum.

**Key questions**

**What do the new findings imply?**

- Among women living most remotely, the MWH model facilitated access to a referral facility when needed, a critical step on the pathway to reduce maternal mortality rates.
- A context appropriate MWH model can be an important component of health system strengthening efforts to address distance as well as quality.
- As countries seek to bend the curve on persisting high rates of maternal and neonatal mortality, MWHs can be an important component in the effort to increase delivery at high quality, advanced facilities in areas where substantial numbers of women live far from health services.

## Introduction

Skilled health personnel attending every birth is an important step toward achieving Target 3.1 of the Sustainable Development Goals (SDG): reducing the global maternal mortality ratio (MMR) to less than 70 deaths per 100 000 live births by 2030.^1^ Though MMR estimates decreased globally by 30% between 1990 and 2015, in 2015 alone over 275 000 women died from causes related to childbirth.^2^ In 2015, sub-Saharan Africa accounted for 48% of all maternal deaths occurring globally.^2^

The WHO recommends use of skilled care at birth and timely access to facilities able to manage complications.^2 3^ Evidence suggests that at the country level, higher facility delivery rates are associated with lower MMRs. While there is an overall increasing trend in facility delivery rates across most low-income and middle-income countries, home delivery remains common among poor, rural populations and populations with low levels of education.^4^ Rural areas of low-income and middle-income countries generally have lower densities of health facilities with the human resources, equipment and medications required to manage obstetric complications. Commonly, rural areas also have poorer populations, less developed road infrastructure and fewer transportation options (ie, public transit), so rural-living women often experience challenges in accessing available, reliable and affordable transportation.^5–8^ During the onset of labour, long distances to fewer capable health facilities over poorer roads using either slow (human or animal-powered) or less affordable (car) transportation options create barriers to rural women accessing skilled delivery care. This is likely particularly acute in the most remote parts of rural areas and in regions with particularly difficult terrain, including mountains and rainy season flooding.

In Zambia, the MMR declined by 30% between 1990 and 2015, but at 247 deaths per 100 000 live births in 2015, it remains well above the SDG global target.^2^ Progress in Zambia has largely been driven by policy changes and large-scale multilateral collaborations such as the Saving Mothers, Giving Life initiative (SMGL).^9^ SMGL employed an intensive and comprehensive set of supply-side and demand-side interventions in targeted districts in Zambia with the aim of improving maternal health outcomes. National health facility delivery rates increased from 44% in 2001/2002 to 84% in 2018, with even greater change observed in SMGL-supported districts.^9 10^ However, women in rural areas continue to face barriers to accessing maternal care for delivery,^11–13^ reflected in the lower facility delivery rate in rural (78.7%) versus urban (93.2%) areas.^10^ Rural-living women in Zambia have discussed the challenges posed by long distances, poor road infrastructure, available and/or unaffordable transportation options, particularly when labour begins at night.^14 15^

Maternity waiting homes (MWHs) are designated spaces near a health facility where women can wait in the weeks before delivery.^16^ The WHO recommended MWHs in 2015, and they have gained traction as a promising intervention that might improve access to intrapartum and postpartum care leading to improved maternal health outcomes.^16–18^ Theoretically, MWHs allow rural-living women to plan their travel more deliberately, potentially via a slower but more affordable transportation method, during the daylight hours and long before the onset of labour. If at the MWH during the onset of labour, skilled care at the health facility would be readily accessible. Additionally, while staying at the MWH, the woman would have additional contacts with the health system through antenatal care (ANC) services; early signs of complications or conditions requiring special attention (eg, twins) are potentially more likely to be identified, allowing for early referral to higher-level care.^19^ We hypothesised that an improved Core MWH Model would increase facility delivery among women living more than 10 km from the health facility in rural Zambia.

Rigorous evidence supporting the efficacy of MWHs is scarce, particularly in sub-Saharan Africa.^3 8 16 20 21^ The Maternity Homes Alliance, a collaboration between the Zambian government and partners, aimed to generate generalisable evidence on the effectiveness of MWHs. Here, the findings of a large-scale quasi-experimental study in SMGL-districts in rural Zambia assessing the impact of an improved Core MWH Model on facility delivery and secondary outcomes related to improved maternal and newborn health among women living most remotely are reported.

## Methods

### Study design and setting

Between March 2016 (baseline) and October 2018 (endline), we implemented a quasi-experimental intervention study in the catchment areas of 40 primarily rural health centres (RHCs) in Choma, Kalomo and Pemba districts in Southern Province, Nyimba and Lundazi districts in Eastern Province, and Mansa and Chembe districts in Luapula Province, Zambia.^22^ A cluster design was selected because of the inherent nature of the intervention. Each cluster was comprised an RHC and its government-defined catchment area households. The targeted districts were part of the SMGL intervention.^9^ We included in the sampling frame all RHCs in the selected districts that met the following eligibility criteria: (1) at least 150 deliveries annually; (2) situated ≤2 hours driving time to the nearest referral hospital capable of providing comprehensive emergency obstetric and neonatal care (CEmONC); and either: (3) able to perform at least five of seven basic emergency obstetric and neonatal care signal functions; or (4) had at least one skilled birth attendant, practiced routine management of third stage labour, and had no stock outs of oxytocin or magnesium sulfate in the previous 12 months. Only referral hospitals offered CEmONC services including parenteral antibiotics, blood transfusions and caesarean sections; the study sites did not.

Prior to the opening of study MWHs, all study districts had received the SMGL initiative (2012–2016) which sought to rapidly reduce maternal mortality through a comprehensive set of interventions to address challenges, and improve maternal health services demand, access and quality.^23^ Among others, interventions included: health facility infrastructure, equipment and medicines stock improvements; training and mentorship of healthcare providers to increase access to EmONC services in the districts; and communication campaigns using community leaders, communication materials, and mass media messaging.^23^

The study districts are primarily rural (67%–95% of the district populations) with pockets of peri-urban centres.^24^ The populations of these districts are generally poor; those living in remote areas have limited access to improved sources of water or sanitation, or to electricity.^25^ At the time of the study described here, the districts had generally similar availability of maternal health services. Facility delivery rates were 56%, 68% and 71% in Southern, Luapula and Eastern Provinces, respectively, according to the Demographic and Health survey conducted prior to the outset of this study.^26^

### Assignment to study arm

The unit of assignment to study arm was the RHC and its catchment area; the unit of analysis was the individual. Of 44 RHCs that met the inclusion criteria, the 40 farthest RHCs were included in the study. Half of the study clusters were assigned prospectively to study arm using a matched-pair randomisation procedure (randomised subgroup).

Study clusters in Choma, Kalomo, Pemba and Nyimba districts were randomised prior to baseline enrolment with equal probability to either the intervention or the control group. Prior to randomisation, catchment areas were matched in pairs first based on government-reported transfer time to nearest CEmONC facility, then best fit to average monthly volume of deliveries over the previous year. Within each matched pair, one catchment area was randomly assigned by the study team to the intervention group using the RAND function in Microsoft Excel. Due to the nature of the intervention, it was not possible to blind participants.

For the other half of study clusters, political considerations precluded random assignment (non-randomised subgroup). Government officials feared community fatigue from so many projects operating in their areas and therefore preferred to identify intervention sites purposefully. Study clusters in Lundazi, Mansa and Chembe districts were non-randomly assigned prior to baseline enrolment. The Ministry of Health assisted in identifying 10 intervention sites. Comparison sites were then identified from eligible RHCs matched on delivery volume and government-reported transport time the nearest CEmONC facility. Sites with a known formal existing MWH structure were not considered in the sample frame; however, baseline data suggested women in the catchment were often accommodated prior to delivery within available bed space or in informal waiting spaces near the health facility, similar to the randomised subgroup.^27^

All intervention clusters received the Core MWH Model (described below) and control clusters implemented a various ‘standard of care’ for women awaiting delivery in Zambia, which included use of a community-constructed structure, women staying informally within RHC wards, and no dedicated space to wait.^17^ Aside from intervention assignment, all study procedures were implemented consistently across the sites.

### Intervention description

The Core MWH Model was developed based on formative research and community input and refined with input from government stakeholders. It aimed to address common barriers to MWH utilisation and to be culturally acceptable.^28 29^ The Core MWH Model included three primary domains. First, all sites had similar infrastructure, equipment and supplies which included concrete floors, latrines, bathing areas, intact roof, storage space, covered cooking space, location near a water supply, lockable doors, cupboards and windows, lighting, beds, bedding, mattresses, mosquito nets and cooking utensils. Each site had a main dormitory for pregnant women and a smaller dormitory for women and newborns, who had just been discharged from the health facility or had returned for a postnatal care visit. Formative research identified the ‘mixing’ of pregnant with newborns as culturally inappropriate; community members requested a separate space within the structure for postpartum women and newborns to stay. Each site also had a private bathing and drying area, per results from the formative work.^28^ Second, each site had a formalised management structure responsible for daily operations and a governance structure with representation from the community, government, traditional leadership and the health centre. Third, all sites were situated within 100 m of, and had formal linkages to, the RHC. Each had health centre staff check in daily on waiting women, and health staff or volunteers offered maternal and child health education courses. Clinical care was conducted at the RHC, not at the MWH. Women learnt about the MWH at ANC visits, through health outreach activities, and through traditional leaders.

Implementation began July 2015; the first intervention Core MWH Model opened in study sites immediately following baseline observation. Intervention MWHs operated for a minimum of 13 months before the endline study.

### Participants

A cross-sectional sample of households was identified at baseline (March–May, 2016) and endline (September–October, 2018) using a multi-stage random sampling procedure. In the first stage, 10 villages located at least 10 km from the RHC from each catchment area were randomly selected with probability proportional to size. To identify eligible villages, every village centre was visited and GPS coordinates were taken to determine travel distance to the RHC along the most direct route, calculated using ArcGIS Online (ESRI, Redlands, California, USA). In the second stage, eligibility was restricted to households with at least one woman 15 years or older who delivered a child in the previous 12 months, irrespective of her place of delivery or current vital status. An exhaustive list of eligible households was created with input from RHC staff, community health volunteers and local traditional leaders. Households were then ordered randomly and visited in that order to confirm eligibility and enrol until the target of approximately six households per village was reached. In the third stage, if a household had more than one eligible woman, one woman was selected at random during enrolment. While we did not ask about previous participation in the survey, it is possible that some women who had another delivery between the data collection rounds were selected for endline. There is no reason to believe this would disproportionately affect one study arm more than the other.

### Procedures

During each round of data collection, a team of trained enumerators who spoke English and the local language spent 6 weeks conducting surveys. Data enumerators were introduced to the household head or senior woman by community volunteers. On confirming household eligibility, the study team consented the household head, geolocated the household and captured demographic information. On completion of this portion of the survey, the eligible woman was selected, consented separately and responded to the remainder of the survey in a private space. Survey topics included household composition and individual characteristics; experience of the last pregnancy including ANC, labour and delivery, birth outcomes and postnatal care; and MWH use. All responses were self-reported; when available, responses were verified against the mother’s antenatal card or the baby’s under-5 card. Data were captured electronically on encrypted tablets using SurveyCTO Collect software (V.2.212; Dobility). Audits on a random selection of 5% of households the following day found few inconsistencies.

### Outcomes

The primary outcome was facility delivery, defined as delivery at either an RHC or a hospital. Women were asked where they delivered their most recent child, including the facility name if applicable. For analysis, responses were dichotomised based on whether the delivery occurred at a health facility or other location.

Secondary outcomes included: (1) use of an MWH while awaiting delivery; (2) maternity care utilisation measures including referral or transfer to a higher-level facility before, during or after labour and attendance at early postnatal care, asked as ‘approximately 3 days after delivery’; (3) hospital-level services received during labour and delivery (parenteral antibiotics, blood transfusion and caesarean section surgery); (4) exposure to counselling services at the time of delivery (family planning, breastfeeding and kangaroo care (ie, early and continuous skin-to-skin contact)); (5) maternal and neonatal vital status after delivery and (6) health behaviours at the time of the interview including use of modern family planning and infant feeding methods. We also include self-reported delivery expenditures in Zambian Kwacha between study arms. Women reported if and how much they had expended on delivery supplies, baby clothes, transportation for delivery, and accommodation while awaiting delivery.^30^

### Sample size calculation

The target sample size for each round of data collection was 2400. We assumed a baseline estimate of 63% facility delivery in rural Zambia, about the average across the provinces where we were working at the time^26^ and an estimated 60 households per cluster. The sample size provided 80% power to detect a 10-percentage point increase in facility delivery due to the intervention and assumed an α of 0.05 and an intracluster correlation coefficient of 0.04.^31^ We did not have the data necessary at the time of conducting the power calculation to make confident estimates of the correlation between baseline and follow-up outcomes for individuals within a cluster. We therefore conservatively assumed this to be zero. When planning for recruitment, we expected approximately 10% refusal. Loss to follow-up was not considered given the repeated cross-section design. The Stata code used for the power calculation was: ‘clustersampsi, binomial beta(0.80) p1(0.63) p2(0.73) k(20) rho(0.04)’.

### Confounders

We constructed a wealth index using household asset information from the broad categories of power source, water source, cooking source, household essentials and luxuries, farming supplies, banking, electronics, and access to internet. The sample was split into quintiles for analysis. We compared potential confounders including characteristics of recently delivered women (age, education, marital status, gravida, parity, antenatal visit, months since delivery and delivery location) and characteristics of the households (wealth quintile, household size, dependency ratio, and distance from village centre to nearest health centre) of the intervention and control groups at both rounds of data collection, including comparisons within randomised and non-randomised subgroups (online supplemental table A1) and characteristics of the sites and MWHs (online supplemental table A2).

10.1136/bmjgh-2021-006385.supp1 Supplementary data

10.1136/bmjgh-2021-006385.supp2 Supplementary data

### Statistical analysis

We determined the impact of the intervention on the primary outcome, facility delivery, for the full sample as well as the randomised and non-randomised subgroups using intention-to-treat analysis. Next, we compared use of MWHs across study groups to better understand intervention uptake. We then estimated the impact of the intervention on the secondary outcomes. We fit a set of generalised estimating equations (GEE) specified as having a binomial distribution for the dependent variable, a logit link function and an exchangeable correlation structure to estimate ORs for all outcomes except for health expenditures. We estimated two separate models to understanding the impact of the intervention on expenditures associated with delivery.^32^ We first estimated a GEE model with the dependent variable indicating whether there was any expenditure on delivery. We then estimated a second GEE model with the dependent variable ln(total expenditure) using only observations with expenditure >0. For this model, we specified a Gaussian distribution for the dependent variable, an identity link function, and an exchangeable correlation structure. For all GEE models, matched-pair was specified as the group variable and robust SEs were estimated using a degrees-of-freedom corrected sandwich estimator. Except for referral from MWH (which had no baseline value prior to intervention), each model included the cluster-level average of the outcomes measured at baseline. Each model also controlled for the variables used in the matching procedure, average monthly volume of deliveries at nearest RHC and transfer time to nearest CEmONC hospital. No additional covariates were included in the main models.

Because half of study clusters were non-randomly assigned, we present adjusted estimates of impact on the primary outcome in online supplemental table A3 using models that included the following covariates: woman’s age (years), education (years), marital status, and primigravida, along with household wealth quintile and distance of the village centre to the nearest government assigned RHC (km). These covariates were selected based on a review of the literature and previous work on where women deliver in Zambia.

10.1136/bmjgh-2021-006385.supp3 Supplementary data

As a robustness check, we also present estimates of impact on the primary outcomes using a set of mixed-effects models that include random effects for matched-pair, health facility catchment area, and village in online supplemental table A4.^33^ Finally, we present estimates of impact on the primary outcomes from a set of generalised linear probability models (ie, GEE specified as having a Gaussian distribution for the dependent variable and an identity link function) in online supplemental table A5. All analyses were conducted using Stata statistical software (StataCorp. 2015. Release V.14). All data for this analysis are publicly available.(dataset)^34^

10.1136/bmjgh-2021-006385.supp4 Supplementary data

10.1136/bmjgh-2021-006385.supp5 Supplementary data

### Patient and public involvement

End-users of the MWHs and other key community-level stakeholders including men, community elders and traditional leadership were involved in conceptualising and designing the intervention during a formative research phase.^28 29^ The intervention design was refined with input from the Ministry of Health. We continued to engage a variety of key stakeholders, including members of the target population, through a rigorous process evaluation that routinely assessed intervention acceptability, and implementation feasibility and fidelity.^19 35 36^

### Consolidated Standards of Reporting Trials statement

We were guided by the Consolidated Standards of Reporting Trials checklist extension for cluster randomised trials in preparing this article.

## Results

### Study population

**Figure 1:**
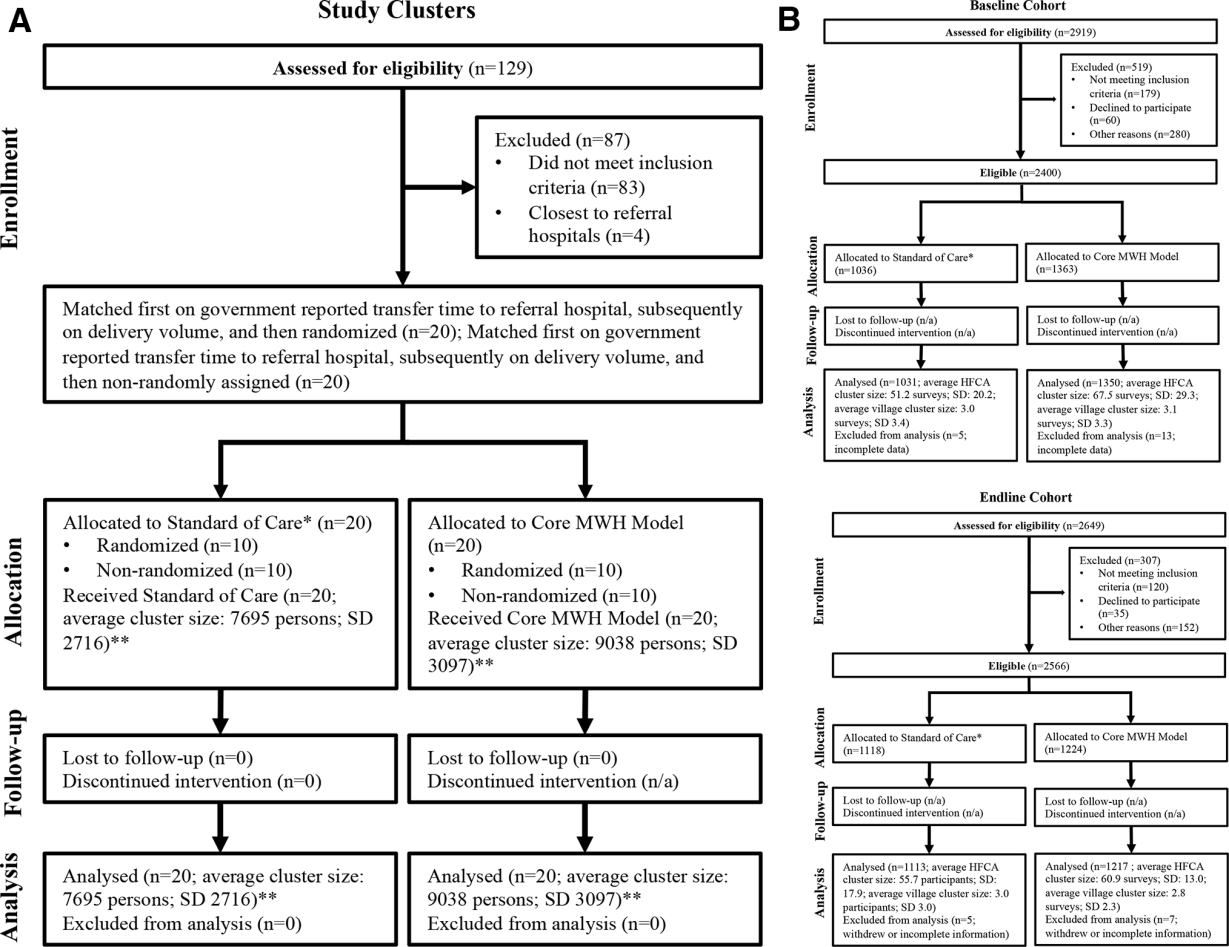
(A) Study profile—study clusters. *Standard of care for women awaiting delivery in rural Zambia included use of a community-constructed structure, women staying informally within rural health centre wards, and no dedicated space to wait. **Cluster size statistics are government reported catchment area population sizes, based on the 2012 List of Health Facilities in Zambia. (B) Study profile—study cohorts. *Standard of care for women awaiting delivery in rural Zambia included use of a community-constructed structure, women staying informally within rural health centre wards, and no dedicated space to wait. MWH, maternity waiting home. HFCA, health facility catchment area.

A total of 40 RHCs were selected and retained in the study; 20 were assigned to the control arm and 20 were assigned to the intervention arm (figure 1A). In the baseline cross-section, 1031 women in control clusters and 1350 women in intervention clusters were interviewed. In the endline cross-section, 1113 women in control clusters and 1217 women in control clusters were interviewed (figure 1B). Over 85% and 90% of eligible households approached participated at baseline and endline, respectively.

Demographic characteristics of respondents were similar in intervention and control groups at both rounds of data collection (table 1) and in both the randomised and non-randomised subgroups (online supplemental table A1). At the time of interview, women were 26 years old on average. Study households were located in villages that were, on average, 14–16 km from their designated RHC. Descriptive characteristics of the RHCs and MWHs were generally similar (online supplemental table A2).

**Table 1:** Household and demographic characteristics of respondents at baseline and endline

	Baseline		Endline	
	Control (n=1031)	Intervention (n=1350)	Control (n=1113)	Intervention (n=1217)
*Characteristics of recently delivered women*				
Age (years), mean (SD)	26.2 (7.1)	26.0 (6.9)	26.0 (6.9)	26.2 (7.0)
Education (years), mean (SD)	5.4 (3.1)	5.2 (3.1)	5.7 (3.2)	5.8 (3.0)
Highest level of education, n (%)				
No education	160 (15.5)	202 (15.0)	152 (13.7)	128 (10.5)
Some primary education	370 (35.9)	598 (44.3)	403 (36.2)	476 (39.1)
Completed primary education	233 (22.6)	243 (18.0)	225 (20.2)	266 (21.9)
Some secondary education	248 (24.1)	284 (21.0)	288 (25.9)	298 (24.5)
Completed secondary education	18 (1.7)	18 (1.3)	35 (3.1)	29 (2.4)
Married/cohabitating, n (%)	890 (86.5)	1202 (89.2)	946 (85.8)	1059 (87.0)
Primigravida, n (%)	219 (21.3)	289 (21.4)	227 (20.4)	260 (21.4)
Gravida, mean (SD)	4.0 (2.6)	3.8 (2.5)	3.7 (2.5)	3.8 (2.4)
Parity, mean (SD)	3.7 (2.4)	3.5 (2.3)	3.4 (2.4)	3.4 (2.4)
Antenatal care visits, n (%)				
None	4 (0.4)	10 (0.7)	6 (0.5)	2 (0.2)
1	24 (2.3)	47 (3.5)	12 (1.1)	13 (1.1)
2	86 (8.3)	131 (9.7)	62 (5.6)	74 (6.1)
3	292 (28.3)	388 (28.7)	233 (20.9)	264 (21.7)
4	376 (36.5)	465 (34.4)	381 (34.2)	414 (34.0)
5+	249 (24.2)	302 (22.4)	417 (37.5)	448 (36.8)
Months since delivery, mean (SD)	5.6 (3.8)	5.6 (3.8)	6.5 (3.8)	6.5 (3.8)
Delivery location of index infant				
At home	156 (15.1)	208 (15.5)	95 (8.5)	82 (6.7)
Rural health centre	720 (69.9)	950 (70.4)	774 (69.5)	859 (70.6)
Referral hospital	123 (11.9)	138 (10.2)	205 (18.4)	251 (20.6)
En route to facility	30 (2.9)	47 (3.4)	39 (3.5)	25 (2.0)
*Household characteristics*				
Poorest wealth quintile, n (%)	164 (16.7)	221 (17.9)	243 (21.9)	220 (18.1)
Household size—persons, median (IQR)	7 (5−9)	6 (4−8)	6 (4−8)	6 (4−8)
Dependency ratio*, mean (SD)	1.5 (0.9)	1.5 (0.8)	1.5 (0.9)	1.4 (0.8)
Distance from village centre to nearest health centre (km), mean (SD)	16.5 (11.9)	14.6 (6.6)	16.4 (13.0)	14.2 (4.3)

*Dependency ratio=ratio of household members under 18 years of age and 65 years of age or over to members aged 18–64 years.

### Impact on facility delivery

Facility delivery at baseline in the intervention and control clusters was similar, at 81% and 82%, respectively (table 1). At endline, 91% of respondents in the intervention cluster delivered at a health facility (RHC or hospital) compared with 88% of control cluster respondents. Effect estimates suggest the intervention had a significant positive impact on the odds of facility delivery at endline in the full sample (OR 1.60 (95% CI 1.13 to 2.27); p=0.008) (table 2). We observed similar results when adjusting for covariates (online supplemental table A3) and when using a set of mixed-effects models that include random effects for matched-pair, health facility catchment area and village (online supplemental table A4). Based on results from a linear probability model (online supplemental table A5), this translates to a 3.5-percentage point increase in facility delivery. Results seem to suggest a stronger effect of the intervention in the randomised subgroup, but the study was not powered to explore this in more detail.

**Table 2:** Impact of the intervention on facility delivery

	Baseline		Endline		Effect estimates	
	Control	Intervention	Control	Intervention	Unadjusted OR (95% CI)	P value
*Full sample*	*(n=1031)*	*(n=1350)*	*(n=1113)*	*(n=1217)*		
Facility delivery, n (%)	843 (81.8)	1088 (80.6)	979 (88.0)	1110 (91.2)	1.60 (1.13 to 2.27)	0.008
*Randomised subgroup*	*(n=598)*	*(n=593)*	*(n=591)*	*(n=619)*		
Facility delivery, n (%)	488 (81.6)	459 (77.4)	502 (84.9)	547 (88.4)	1.84 (1.13 to 3.01)	0.015
*Non-randomised subgroup*	*(n=1031)*	*(n=1350)*	*(n=1113)*	*(n=1217)*		
Facility delivery, n (%)	355 (82.0)	629 (83.6)	477 (91.4)	563 (94.2)	1.35 (0.72 to 2.52)	0.346

All models include as covariates: the variables used in the matched randomisation procedure (average monthly volume of deliveries at the nearest health centre and transfer time to comprehensive emergency obstetric and neonatal care hospital) and the cluster-average value of the outcome at baseline.

### Impact on secondary outcomes

At endline, 48% of women in the intervention cluster reported staying at an MWH while awaiting delivery as compared with 26% of women in the control cluster (table 3). Women in the intervention group stayed an average of 16.0 days (SD 18.9) and women in the control group stayed an average of 11.4 days (SD 14.1) (p<0.02) while awaiting delivery.

**Table 3:** Impact of the intervention on secondary outcomes

	Baseline		Endline		Effect estimate	
	Control N=1031 n (%)	Intervention N=1350 n (%)	Control N=1113 n (%)	Intervention N=1217 n (%)	Unadjusted OR (95% CI)	P value
*Healthcare utilisation*						
Used maternity waiting home while awaiting delivery	270/1031 (26.2)	378/1350 (28.0)	292/1113 (26.2)	583/1217 (47.9)	2.44 (1.62, 3.67)	<0.001
Referred or transferred to a hospital during pregnancy or delivery	67/1031 (6.5)	79/1345 (5.9)	73/1113 (6.6)	101/1217 (8.3)	1.35 (0.94 to 1.93)	0.106
Postnatal care within 3 days	108/1030 (10.5)	163/1345 (12.1)	154/1108 (13.9)	268/1209 (22.2)	1.55 (1.10 to 2.19)	0.013
*Hospital-level services received during labour and delivery*						
Intravenous antibiotics	315/1028 (30.6)	375/1337 (28.1)	378/1091 (34.7)	472/1185 (39.8)	1.35 (0.92 to 1.99)	0.124
Blood transfusion	29/1023 (2.8)	53/1341 (4.0)	43/1100 (3.9)	70/1204 (5.8)	1.62 (0.92 to 2.87)	0.095
Caesarean section surgery	33/1031 (3.2)	47/1350 (3.5)	44/1110 (4.0)	84/1214 (6.9)	1.71 (1.16 to 2.54)	0.007
*Counselling received around time of delivery*						
Family planning	548/1022 (53.6)	729/1335 (54.6)	727/1097 (66.3)	880/1193 (73.8)	1.48 (1.15 to 1.91)	0.002
Breastfeeding	579/1028 (56.3)	768/1337 (57.4)	720/1097 (65.6)	876/1194 (73.4)	1.51 (1.20 to 1.90)	<0.001
Kangaroo care	363/1015 (35.8)	513/1312 (39.1)	604/1090 (55.4)	762/1173 (65.0)	1.44 (1.15 to 1.79)	0.001
*Health behaviours reported at time of the survey*						
Currently using modern family planning method	337/1031 (32.7)	440/1348 (32.7)	526/1111 (47.3)	608/1216 (50.0)	1.13 (0.91 to 1.40)	0.267

All models include as covariates: the variables used in the matched randomisation procedure (average monthly volume of deliveries at the nearest health centre and transfer time to comprehensive emergency obstetric and neonatal care hospital) and the cluster-average value of the outcome at baseline.

The Core MWH Model was associated with increased odds of using an MWH while awaiting delivery (OR 2.44 (1.62 to 3.67); p<0.001), attending postnatal care within 3 days of delivery (OR 1.55 (1.10 to 2.19); p=0.013) and with receipt of critical services offered only at referral hospitals caesarean section surgery (OR 1.71 (1.16 to 2.54); p=0.007) (table 3).

The intervention was also associated with increased exposure to counselling for family planning (OR 1.48 (1.15 to 1.91); p=0.002), breastfeeding (OR 1.51 (1.20 to 1.90); p<0.001) and kangaroo care (OR 1.44 (1.15 to 1.79); p=0.001). However, we found the intervention had no impact on the odds of using modern family planning methods at the time of the survey and over 99% of women reported they were currently breastfeeding their child. Data on maternal death was limited to the day of delivery. There were no reported maternal deaths on the day of delivery in either study arm at baseline; there was one reported maternal death on the day of delivery in the intervention arm at endline. There was no difference in reported stillbirths or infant death on the day of delivery at baseline (14 control, 13 intervention) or endline (17 control, 19 intervention).

Finally, we observed no significant difference between groups on the odds of having spent money on labour and delivery (OR 0.95 (0.57 to 1.59); p=0.852) nor on the amount spent for those who did spend something (table 4).

**Table 4:** Impact of the intervention on health expenditures for delivery

	Baseline		Endline		Effect estimate	
	Control n (%)	Intervention n (%)	Control n (%)	Intervention n (%)	Unadjusted OR (95% CI)	P value
Any expenditure	1005/1031 (97.5)	1313/1350 (97.3)	1070/1113 (96.1)	1167/1217 (95.9)	0.95 (0.57 to 1.59)	0.852
	**Control median (IQR**)	**Intervention median (IQR**)	**Control median (IQR**)	**Intervention median (IQR**)	β* (**95% CI**)	**P value**
Total expenditure if >0 (ZMK)	260 (179–380)	240 (154–355)	372 (250–520)	365 (250–523)	0.04 (−0.08 to 0.15)	0.504

All models include as covariates: the variables used in the matched randomization procedure (average monthly volume of deliveries at the nearest health centre and transfer time to comprehensive emergency obstetric and neonatal care hospital) and the cluster-average value of the outcome at baseline.

*Based on model with ln(total expenditure) as dependent variable.

## Discussion

Our study aimed to rigorously test the relationship between an improved MWH model and facility delivery among women living farthest from health centres in rural Zambia. Our findings add to the evidence base on the impact of MWH interventions on access to maternity care. In sub-Saharan Africa, long distances to health facilities are frequently reported as barriers to accessing maternity care^7 8^ and one recent meta-analysis of studies from the region found that distance from maternity care is inversely correlated with utilisation of maternity services.^6 7^ The Core MWH Model tested here significantly increased facility delivery among women living at least 10 km from their designated RHCs which met our inclusion criteria, directly addressing this distance barrier.

Additionally, there were consistent increases in the intervention arm of approximately 2 percentage points in the proportion of women delivering at a CEmONC referral hospital and similar increases in the proportion of women who received important CEmONC services including caesarean section surgery. Findings suggest that, among women living most remotely, the MWHs might facilitate access to a referral centre when needed. These findings are consistent with previously reported implementation data from the study wherein health facility staff at intervention sites qualitatively reported that the MWHs gave them more time to observe and appropriately manage and refer complicated cases in a timely manner.^19^ Additionally, findings are consistent with recently published data from the concurrent implementation evaluation of this study which found, when looking at all health facility and MWH utilisation data in these same sites, similar increases in facility delivery and referral for hospital care among women living >10 km from the health facility, those with historically low access.^37^ In low-resource settings where RHCs may not be equipped to manage complications, timely identification and referrals are critical. Findings from these two studies suggest MWHs may facilitate that access, with no difference in self-reported expenditures.

The WHO recommendation for MWH use assumes that by introducing MWHs, a country can increase facility-based births, thereby reducing obstetric complications, maternal deaths and perinatal mortality. Past studies have shown associations between MWH use and these health outcomes,^18 38^ but the causal pathway, through increasing facility-based delivery, has not been proven using rigorous methods. Our study was powered on facility delivery, an important intermediate process indicator on the causal pathway to improving maternal health outcomes.

The evidence around the effectiveness of MWHs as an intervention to increase facility delivery has been largely observational or qualitative and mostly based on facility-level data.^16 20^ The impact on facilitating access for remote-living women is scarce; only one prestudy and poststudy in Timor-Leste assessed distance and found MWHs did not improve facility delivery for remote-living women.^39^ Additionally, to the best of our knowledge, the only randomised trial evaluating the impact of MWHs on facility delivery was conducted recently in a more densely populated setting in Ethiopia. This three-armed trial found that improved MWHs with local leader training, and local leader training alone (without MWH improvements), increased facility delivery compared with usual care, though neither result was statistically significant likely because of low uptake of the intervention.^40^ Adding to the current literature, our large-scale intervention study uses population-level data from seven rural Zambian districts to show that MWHs are an effective intervention to improve facility delivery in rural areas, providing some of the first empirical estimates that have long been missing in the argument for MWH expansion.

It is important to note that our sites were deliberately selected from SMGL-supported districts to ensure the health facilities had the capacity to manage any increased demand generated by the MWH. To further ensure this, our site-level inclusion criteria limited the sites most experienced—indicated by high delivery volume—and equipped to manage basic complications. Results should be interpreted within this context as it is possible we may not have observed such improved access to facility delivery if the health facilities themselves were not perceived to be of high quality. Careful consideration should be given to the placement of future MWHs in any context.

Similar to the perception of the health facility, an important factor associated with MWH use is women’s perceptions of MWH quality.^41^ Formative research found that if MWHs were to be used in this context, they must be considered by the community to be safe, comfortable and culturally appropriate, and include a space for postnatal women to be separate from pregnant women.^28 29^ The high intervention uptake observed in this study—a near doubling of MWH utilisation in the intervention arm—and the increase in postnatal care attendance suggests that the intervention design was acceptable. The separate space designated for postnatal women allowed women who had been discharged a place to stay until the 3-day postnatal visit. Women were also informed they could return and use the space for any follow-up postnatal visits. Though we observed a near doubling of the MWH utilisation, slightly less than half of respondents reported using an MWH while awaiting delivery. There are multiple reasons why women might not use an MWH prior to delivery. For example, women often stay temporarily with family, some have better access to transportation options, and others have home responsibilities that preclude them from leaving prior to the onset of labour. This should be explored in more detail as there is an opportunity to increase utilisation even further.

Additionally, consistent with our observed increase in exposure to maternal and well-baby counselling services, at intervention facilities, health staff checked in daily on waiting women, and staff or community health workers provided health education and counselling in a communal space.^19^ These frequent interactions with health staff and continuous exposure to health education sessions over the course of the stay may have a broader influence on maternal and neonatal health outcomes compared with short stays in a health facility during delivery. This is consistent with other evidence showing associations with women’s groups and improved health outcomes.^42 43^

Findings from this study are encouraging in terms of a potential strategy to increase access to facility delivery and maternity care for women living most remotely. An increase of 3.5 percentage points may seem modest, particularly in light of other interventions aiming to improve access to maternity care. Conditional cash transfers, for example, are promising, having been shown to increase caesarean section rates among the rural poor in Mexico,^44^ to increase facility delivery by 41% in Nigeria,^45^ and are currently being studied for impact on the continuum of maternity care in a large-scale cluster randomised controlled trial in Kenya.^46^ It is difficult to compare given that none of the cash transfer studies specifically address distance, but when interpreted in the context of the unexpectedly high baseline facility delivery rates (>80%) and the particularly remote population, the 3.5 percentage points may still be programmatically meaningful. Understanding the impact of different interventions on comparable populations and the cost-effectiveness of each are important next steps.

MWHs are relevant to national and global policy on access to facility delivery. The Zambian government is committed to improving facility delivery as evidenced by a general trend of increasing rates and the high baseline delivery rates observed in this study.^12^ Investment in MWHs offers an opportunity to continue improving facility delivery rates by reaching the most remote women. However, the location of an MWH must be carefully considered, in light of the site inclusion criteria in this study. It could be potentially harmful to increase utilisation at health facilities unequipped to manage basic obstetrical complications and make timely referrals to higher level care. Some experts have suggested that redesigning delivery care systems to emphasise high volume/high-capacity sites—primarily hospitals—would greatly improve quality of care and health outcomes.^47^ This, however, would likely require women to travel further to care and encounter greater access barriers, particularly in terms of distance. In the future, strategically placed MWHs could help realise this vision for reorganising maternity care.

### Limitations

While findings from this study provide rigorous empirical evidence on the impact of MWHs in this setting, this study has several limitations. First, political considerations required adaption of the study design, resulting in randomised and non-randomised subgroups which may result in residual confounding in the overall sample. Second, there is a potential for recall bias, as the survey asked women to discuss their most recent delivery up to 12 months prior. To mitigate this, we confirmed responses with the mother’s health card or baby’s under-5 card when available and limited questions to major events during delivery that were more likely to be remembered (ie, caesarean section). We do not expect recall bias to disproportionately affect one group. Third, this impact evaluation collected population-level data from women living in villages located more than 10 km from the catchment area RHC. This limits our ability to understand the impact of the Core MWH Model on those living closer. Fourth, this study focused on healthcare utilisation, which is a process indicator and not a health outcome. Future studies should address whether higher facility utilisation driven by MWHs ultimately translates into mortality decreases for mothers and newborns. Mortality reductions will critically depend on selecting facilities that can provide life-saving services consistently and with high quality. Fifth, we did not account for the loss in degrees-of-freedom resulting from the matched-pair design in the original power calculation and, as a result, may have slightly underestimated the needed sample size. Finally, our study districts were specifically chosen because of the intensive package of supply and demand-side interventions provided through SMGL to ensure health facility capacity to meet potentially increased demand. Our inclusion criteria only allowed consideration of health facilities within 2 hours travel time from a referral hospital to ensure timely transfer of women experiencing complications was feasible, limiting the generalisability of our findings to similar contexts. Health facilities of lesser quality and capacity, or substantially further from higher-level care, may not experience the same results from having an MWH. Overall, results should be interpreted in the context of the Zambian health system and health policies.

## Conclusion

The Core MWH Model offers remote, rural Zambian women increased access to skilled birth attendance at equipped health facilities. The intervention increased hospital delivery and improved exposure to counselling support. For women experiencing the combined barriers of distance and limited or expensive transport options, MWHs can be a solution to connect with the formal health system. Looking to the future, as countries seek to bend the curve on persisting high rates of maternal and newborn mortality, MWHs can be an important component in the effort to increase delivery at high quality, advanced facilities in areas where substantial numbers of women live far away.

## Data Availability

Data are available in a public, open access repository. De-identified data for this analysis will be made available through OpenBU. The OpenBU repository has data access policies and procedures consistent with NIH data sharing policies.
