# Supplementary material for: If we build it, will they come? Results of a quasi-experimental study assessing the impact of maternity waiting homes on facility-based childbirth and maternity care in Zambia
Source: BMJ Glob Health. 2021 Dec 6;6(12):e006385. doi: 10.1136/bmjgh-2021-006385 (PMC8655557; doi:10.1136/bmjgh-2021-006385)
Supplement: Supplementary data [file bmjgh-2021-006385supp001.pdf]

Table A1. Characteristics of study participants in randomised and non-randomised subgroups

	Baseline			Endline		
**Panel 1· Randomised subgroup**	**Control (n=598)**	**Intervention (n=594)**	**p-value**	**Control (n=591)**	**Intervention (n=619)**	**p-value**
Characteristics of recently delivered women						
Age (years), *mean (SD)*	26·2 (7·3)	25·9 (6·7)	0·51	26·0 (6·9)	26·4 (7·0)	0·22
Education (years), *mean (SD)*	5·9 (3·1)	5·8 (3·2)	0·81	6·4 (3·3)	6·3 (3·0)	0·86
Highest level of education *n (%)*						
No education	81 (13·5)	83 (14·0)	0·92	70 (11·8)	63 (10·2)	0·96
Some primary education	172 (28·8)	199 (33·5)		162 (27·4)	190 (30·7)	
Completed primary education	167 (27·9)	133 (22·4)		139 (23·5)	160 (25·8)	
Some secondary education	166 (27·8)	163 (27·4)		189 (32·0)	183 (29·6)	
Completed secondary education	10 (1·7)	14 (2·4)		29 (4·9)	20 (3·2)	
Married/cohabitating, *n (%)*	494 (82·9)	511 (86·3)	0·14	481 (81·4)	522 (84·3)	0·34
Primigravida, *n (%)*	132 (22·1)	116 (19·5)	0·28	126 (21·3)	122 (19·7)	0·53
Gravida, *mean (SD)*	4·0 (2·6)	3·9 (2·4)	0·49	3·8 (2·5)	3·9 (2·4)	0·32
Parity, *mean (SD)*	3·8 (2·5)	3·6 (2·3)	0·58	3·4 (2·4)	3·6 (2·5)	0·57
Antenatal care visits, *n (%)*						
None	1 (0·2)	3 (0·5)	0·19	3 (0·5)	1 (0·2)	0·97
1	14 (2·3)	29 (4·9)		4 (0·7)	7 (1·1)	
2	46 (7·7)	62 (10·4)		34 (5·8)	31 (5·0)	
3	168 (28·1)	163 (27·4)		127 (21·5)	126 (20·4)	
4	227 (38·0)	186 (31·3)		205 (34·7)	229 (37·0)	
>4	142 (23·7)	148 (24·9)		216 (36·5)	225 (36·4)	
Months since delivery, *mean (SD)*	5·6 (3·9)	5·6 (3·8)	0·96	6·6 (3·6)	6·6 (3·7)	0·99
Delivery location of index infant, *n (%)*						
Home	97 (16·2)	114 (19·2)	0·77	72 (12·2)	54 (8·7)	0·71
Rural Health Center	396 (66·2)	386 (65·1)		383 (64·8)	425 (68·7)	
Referral Hospital	93 (15·5)	74 (12·5)		119 (20·1)	122 (19·7)	
En route to facility	12 (2·0)	19 (3·2)		17 (2·9)	18 (2·9)	
Household characteristics						
Poorest wealth quintile, *n (%)*	44 (7·6)	43 (7·4)	0·97	45 (7·6)	42 (6·8)	0·73
Dependency ratio, *mean (SD)*	1·6 (0·9)	1·6 (0·9)	0·64	1·6 (0·9)	1·5 (0·8)	0·81
Distance from nearest facility (km), *mean (SD)*	15·2 (7·1)	15·3 (5·1)	0·94	15·0 (6·9)	15·5 (4·8)	0·81
**Panel 2· Non-randomised subgroup**	**Control (n=433)**	**Intervention (n=756)**	**p-value**	**Control (n=522)**	**Intervention (n=598)**	**p-value**
Characteristics of recently delivered women						

Age (years), *mean (SD)*	26·2 (6·9)	26·1 (7·0)	0·78	26·0 (6·9)	26·0 (6·9)	0·95
Education (years), *mean (SD)*	4·8 (3·1)	4·7 (2·9)	0·85	5·1 (3·0)	5·3 (2·9)	0·55
Highest level of education, *n (%)*						
No education	79 (18·2)	119 (15·7)	0·67	82 (15·7)	65 (10·9)	0·77
Some primary education	198 (45·7)	399 (52·8)		241 (46·2)	286 (47·8)	
Completed primary education	66 (15·2)	110 (14·6)		86 (16·5)	106 (17·7)	
Some secondary education	82 (18·9)	121 (16·0)		99 (19·0)	115 (19·2)	
Completed secondary education	8 (1·9)	4 (0·5)		6 (1·1)	9 (1·5)	
Married/cohabitating, *n (%)*	396 (81·5)	691 (91·5)	0·98	465 (89·1)	537 (89·8)	0·55
Primigravida, *n (%)*	87 (20·1)	173 (22·9)	0·27	101 (19·4)	138 (23·1)	0·26
Gravida, *mean (SD)*	3·9 (2·6)	3·8 (2·5)	0·50	3·7 (2·4)	3·6 (2·3)	0·56
Parity, *mean (SD)*	3·6 (2·4)	3·4 (2·3)	0·35	3·3 (2·3)	3·2 (2·3)	0·40
Antenatal care visits, *n (%)*						
None	3 (0·7)	7 (0·9)	0·99	3 (0·6)	1 (0·2)	0·59
1	10 (2·3)	18 (2·4)		8 (1·5)	7 (1·1)	
2	40 (9·2)	69 (9·1)		28 (5·4)	31 (5·0)	
3	124 (28·6)	225 (29·8)		106 (20·3)	126 (20·4)	
4	149 (34·4)	279 (36·9)		176 (33·7)	229 (37·0)	
>4	107 (24·7)	154 (20·4)		201 (38·5)	225 (36·4)	
Months since delivery, *mean (SD)*	5·4 (3·7)	5·5 (3·7)	0·89	6·4 (3·9)	6·5 (3·9)	0·86
Delivery location of index infant, *n (%)*						
Home	59 (13·6)	94 (12·5)	0·93	23 (4·4)	28 (4·7)	0·77
Rural health center	325 (75·1)	566 (75·3)		391 (74·9)	434 (72·6)	
Referral hospital	30 (6·9)	64 (8·5)		86 (16·5)	129 (21·6)	
En route to facility	18 (4·2)	28 (3·7)		22 (4·2)	7 (1·2)	
Household characteristics						
Poorest wealth quintile, *n (%)*	120 (27·7)	178 (23·5)	0·41	198 (37·9)	178 (29·8)	0·16
Dependency ratio, *mean (SD)*	1·4 (0·9)	1·4 (0·8)	0·26	1·4 (0·9)	1·3 (0·8)	0·20
Distance from nearest facility (km), *mean (SD)*	18·4 (16·3)	14·0 (7·6)	0·34	17·9 (17·4)	12·8 (3·1)	0·30
