# Supplementary material for: If we build it, will they come? Results of a quasi-experimental study assessing the impact of maternity waiting homes on facility-based childbirth and maternity care in Zambia
Source: BMJ Glob Health. 2021 Dec 6;6(12):e006385. doi: 10.1136/bmjgh-2021-006385 (PMC8655557; doi:10.1136/bmjgh-2021-006385)
Supplement: Supplementary data [file bmjgh-2021-006385supp002.pdf]

Table A2. Characteristics of study clusters in randomised and non-randomised subgroups

	Randomised subgroup		Non-randomised subgroup		All Study Sites	
	Control N=10	Intervention N=10	Control [k] N=10	Intervention N=10	Control [k] N=20	Intervention N=20
Health Facility Characteristics [a]						
Deliveries (annual), mean (SD) [b]	282.2 (82.6)	283.0 (139.5)	261.2 (138.8)	286.8 (190.8)	271.7 (111.7)	284.9 (162.7)
BEmONC Signal Functions, mean (SD) [c]	3.9 (1.8)	4.4 (1.2)	3.9 (1.2)	3.4 (2.0)	3.9 (1.5)	3.9 (1.7)
Distance to nearest referral hospital (km), mean (SD) [d]	36.2 (18.2)	44.0 (16.2)	31.7 (15.7)	36.6 (15.5)	34.0 (16.7)	40.3 (15.9)
Drive time to nearest referral hospital (minutes), mean (SD) [e]	58.0 (44.3)	66.9 (41.5)	33.5 (14.7)	35.0 (13.7)	45.8 (34.5)	51.0 (34.4)
Qualified for study under selection criteria A, n (%) [f]	4 (40.0)	6 (60.0)	4 (40.0)	3 (30.0)	8 (40.0)	9 (45.0)
Qualified for study under selection criteria B, n (%) [g]	6 (60.0)	4 (40.0)	6 (60.0)	7 (70.0)	12 (60.0)	11 (55.0)
Catchment Area Characteristics [h]						
Number of villages, mean (SD)	46.1 (22.9)	48.9 (16.7)	54.9 (18.5)	64.5 (37.0)	50.5 (20.8)	56.7 (29.1)
Catchment area population, mean (SD)	12,437.9 (4666.4)	10,928.8 (3,189.9)	8,729.2 (3,346.3)	8,812.1 (4,155.2)	10,485.9 (4,347.0)	9,814.7 (3,786.1)
Catchment area population >10km, mean (SD)	3,838.4 (2,303.7)	4,179.3 (2,460.3)	1,665.4 (1,038.8)	1,887.9 (829.0)	2,751.9 (2,062.8)	3,101.0 (2,171.9)
Distance of furthest village in catchment area from assigned health facility (km), mean (SD)	20.3 (9.1)	23.1 (8.7)	27.5 (22.7)	18.4 (5.1)	23.9 (17.2)	20.7 (7.3)
MWH Characteristics at Start of Intervention [i]						
Roof, n (%)	7 (70.0)	10 (100.0)	n/a [k]	10 (100.0)	7 (35.0)	20 (100.0)
Concrete walls, n (%)	3 (30.0)	10 (100.0)	n/a [k]	10 (100.0)	3 (15.0)	20 (100.0)
Beds, mean (SD)	2.9 (3.2)	14 (0)	n/a [k]	20 (0)	1.5 (2.6)	17.0 (3.1)
Reliable light source, n (%)	4 (40.0)	10 (100.0)	n/a [k]	10 (100.0)	4 (20.0)	20 (100.0)
Available latrines or toilets, n (%)	8 (80.0)	10 (100.0)	n/a [k]	10 (100.0)	8 (40.0)	20 (100.0)
Private shower/bathing area, n (%)	5 (50.0)	10 (100.0)	n/a [k]	10 (100.0)	5 (25.0)	20 (100.0)
Designated cooking area, n (%)	8 (80.0)	10 (100.0)	n/a [k]	10 (100.0)	8 (40.0)	20 (100.0)
Dedicated postnatal care room, n (%)	0 (0)	10 (100.0)	n/a [k]	10 (100.0)	0 (0)	20 (100.0)
Annual women waiting, mean (SD) [j]	110.9 (67.2)	187.5 (97.4)	n/a [k]	196.2 (118.1)	55.5 (73.3)	191.9 (105.5)

MWH = maternity waiting home; km = kilometres; SMGL = Saving Mothers Giving Life initiative; BEmONC = basic emergency obstetric and neonatal care; CEmONC = comprehensive emergency obstetric and neonatal care

[a] These data were used for selecting and matching the sites.

[b] Delivery volume data are from the Zambian health information management system (HIMS) for 2014.

[c] BEmONC signal function data are from the SMGL Facility Assessments 2015. There was missing signal function data for two study sites.

[d] Distance data were extracted from SMGL Health Facility Assessments 2015 for the non-randomised sub-group and calculated using ArcGIS® Online for randomised subgroup.

[e] Time data were extracted from SMGL Health Facility Assessments 2015

[f] Selection criteria A: 1) at least 150 deliveries annually (actual 2014 or projected by SMGL for 2015); 2) situated ≤2 hours driving time to the nearest referral hospital capable of providing CEmONC; and 3) able to perform at least 5 of 7 BEmONC signal functions. If a study site qualified under both criteria, it was only counted for criteria A here.

[g] Selection criteria B: 1) at least 150 deliveries annually (actual 2014 or projected by SMGL for 2015); 2) situated ≤2 hours driving time to the nearest referral hospital capable of providing CEmONC; 3) had at least one skilled birth attendant; 4) practiced routine management of third stage labor; and 5) had no stock outs of oxytocin or magnesium sulfate in the previous 12 months. Sites that did not meet Criteria B5 in the SMGL Facility Assessment 2015 data were reassessed in mid-2015 by the study team; the district health office and SMGL teams ensured there would be no stockouts after implementation of the MWHs.

[h] Data were collected by study partners from village leaders or the district health office. Distance and population data were collected in 2016 prior to the baseline and amended at endline.

[i] Data were collected by study partners using a checklist derived from the Core MWH Model. Data are from three months after the opening of each intervention site for the intervention site and its matched pair.

[j] Data collected by study partners using an MWH register. Data are for the last 12 months of study implementation (August 2017 – July 2018).

[k] The control sites for the non-randomised subgroup reported no physical space for women to wait at the health facility for delivery.
