# Supplementary material for: If we build it, will they come? Results of a quasi-experimental study assessing the impact of maternity waiting homes on facility-based childbirth and maternity care in Zambia
Source: BMJ Glob Health. 2021 Dec 6;6(12):e006385. doi: 10.1136/bmjgh-2021-006385 (PMC8655557; doi:10.1136/bmjgh-2021-006385)
Supplement: Supplementary data [file bmjgh-2021-006385supp003.pdf]

Table A3: Impact of the intervention on facility delivery adjusting for covariates

	Effect estimate	
	**aOR (95% CI)**	**p-value**
**Full sample**		
Facility delivery	1·61 (1·11, 2·35)	0.012
**Randomised subgroup**		
Facility delivery	1·91 (1·17, 3·11)	0·009
**Non-randomised subgroup**		
Facility delivery	1·35 (0·73, 2·51)	0·340

aOR = adjusted odds ratio; CI = Confidence Interval
Models include the following covariates: the matching variables (average volume of deliveries at nearest health center and transfer time to nearest CEmONC referral hospital), the baseline value of the outcome, woman's age, education, marital status, and primigravida status; household wealth and distance to the health centre·
