# Supplementary material for: If we build it, will they come? Results of a quasi-experimental study assessing the impact of maternity waiting homes on facility-based childbirth and maternity care in Zambia
Source: BMJ Glob Health. 2021 Dec 6;6(12):e006385. doi: 10.1136/bmjgh-2021-006385 (PMC8655557; doi:10.1136/bmjgh-2021-006385)
Supplement: Supplementary data [file bmjgh-2021-006385supp004.pdf]

Table A4: Impact of the intervention on facility delivery using a mixed-effects model

	ICC			Effect estimate	
	**Matched-pair**	**HFCA | matched-pair**	**Village | HFCA | matched-pair**	**Unadjusted OR (95% CI)**	**p-value**
**Full sample**					
Facility delivery	0.04	0·05	0.24	1·68 (1·09, 2·58)	0.019
**Randomised subgroup**					
Facility delivery	0.05	0·06	0.25	1·87 (1·15, 3·03)	0·012
**Non-randomised subgroup**					
Facility delivery	0.00	0·01	0.15	1·44 (0·71, 2·92)	0·317

ICC = Intracluster Correlation Coefficient; HFCA = health facility catchment area OR = Odds Ratio; CI = Confidence Interval
Models include random effects for matched-pair, HFCA, and village.
Models include the following covariates: the matching variables (average volume of deliveries at nearest health centre and transfer time to nearest CEmONC referral hospital) and the baseline value of the outcome.
