# Supplementary material for: If we build it, will they come? Results of a quasi-experimental study assessing the impact of maternity waiting homes on facility-based childbirth and maternity care in Zambia
Source: BMJ Glob Health. 2021 Dec 6;6(12):e006385. doi: 10.1136/bmjgh-2021-006385 (PMC8655557; doi:10.1136/bmjgh-2021-006385)
Supplement: Supplementary data [file bmjgh-2021-006385supp005.pdf]

Table A5. Impact of the intervention on primary and secondary outcomes using a set of generalized linear probability models

	**β (95% CI)**	**p-value**
**Primary outcome**		
Facility delivery	0.035 (0.003, 0.069)	0.034
**Secondary outcomes**		
Health care utilization		
Utilized maternity waiting home while awaiting delivery	0.197 (0.118, 0.277)	<0.001
Referred or transferred to a hospital during pregnancy or delivery	0.021 (-0.004, 0.046)	0.105
Postnatal care within 3 days	0.067 (0.018, 0.115)	0.007
Hospital-level services received during labor and delivery		
Intravenous antibiotics	0.070 (-0.019, 0.158)	0.122
Blood transfusion	0.022 (-0.003, 0.047)	0.084
Caesarean section surgery	0.030 (0.010, 0.049)	0.003
Counselling received around time of delivery		
Family planning	0.082 (0.030, 0.133)	0.002
Breastfeeding	0.087 (0.037, 0.136)	0.001
Kangaroo care	0.086 (0.033, 0.138)	0.001
Health behaviors reported at time of the survey		
Currently using modern family planning method	0.030 (-0.023, 0.082)	0.266

Models include the following covariates: the matching variables (average volume of deliveries at nearest health centre and transfer time to nearest CEmONC referral hospital) and the baseline value of the outcome.
